# Radiological presence of vascular loops in the cerebellopontine angle region in patients with unilateral Ménière’s disease

**DOI:** 10.1007/s00405-023-07838-9

**Published:** 2023-02-03

**Authors:** Ping Lei, Kaijun Xia, Jing Li, Yingzhao Liu, Renhong Zhou, Jingjing Liu, Hongchang Wang, Yue Zhou, Yangming Leng, Bo Liu

**Affiliations:** 1 Department of Radiology, Union Hospital, Tongji Medical College, Huazhong University of Science and Technology, Wuhan, 430022 China; 2 Department of Otorhinolaryngology, Union Hospital, Tongji Medical College, Huazhong University of Science and Technology, Wuhan, 430022 China

**Keywords:** Ménière’s disease (MD), Cerebellopontine angle (CPA), Internal auditory canal (IAC), Anterior inferior cerebellar artery (AICA), Vascular loop, Magnetic resonance imaging (MRI)

## Abstract

**Objective:**

The relationship between vascular compression of the vestibulocochlear nerve and audio-vestibular symptoms remains controversial. We aimed to examine the radiological features of vascular loops signs in cerebellopontine angle (CPA) and internal auditory canal (IAC) in patients with unilateral Ménière’s disease (MD).

**Methods:**

One hundred and thirty-seven patients with unilateral definite MD and 69 control subjects (138 ears) were enrolled. All subjects received magnetic resonance imaging of CPA-IAC. The configuration of vascular loops in CPA-IAC, based on the Kazawa classification system, from MD-affected, non-affected and control ears were compared. The associations between imaging findings and Ménière’s stage, electrocochleogram (EcochG) and caloric test were analyzed.

**Results:**

(1) Among the MD-affected ears, 6 cases (4.4%) were classified as Kazawa type IA, 27 cases (19.7%) as IB, 60 cases (43.8%) as IIA, and 44 cases (32.1%) as IIB. No significant interaural difference in the distribution of Kazawa's types was found (${x}^{2}$ = 4.737, *p* = 0.578) in unilateral MD patients. (2) The distribution of Kazawa's types were not significantly different between the MD-affected ears and the control subjects (${x}^{2}$ = 2.876, *p* = 0.411). (3) No relationship was found between Kazawa staging of the MD-affected ear and Ménière’s stage (*H* = 2.679, *p* = 0.444), EcochG (${x}^{2}$ = 0.827, *p* = 0.867) and caloric test (${x}^{2}$ = 4.116, *p* = 0.248).

**Conclusions:**

In patients with unilateral MD, the configuration of vascular loops in CPA-IAC region, measured by Kazawa criteria, did not correlate with the laterality, clinical stage, the results of EcochG and caloric test, suggesting that vascular loops may be natural anatomical variations for patients with MD.

**Supplementary Information:**

The online version contains supplementary material available at 10.1007/s00405-023-07838-9.

## Introduction

Schultze in 1875 described a case of hemifacial spasm arising from compression of the facial nerve by a vertebral artery aneurysm, which was the first report of vascular compression of the cranial nerve. In 1934, Dandy first reported a case of trigeminal neuralgia secondary to trigeminal nerve compression [1]. Jannetta introduced the concept of neurovascular compression in 1975 to explain the pathogenesis of trigeminal neuralgia and performed the first microvascular decompression of the cranial nerve in a patient with this condition [2]. Until now, although vascular compression is widely accepted as a cause of trigeminal neuralgia and hemifacial spasm, there is still controversy regarding vascular compression of the vestibulocochlear nerve resulting in audio-vestibular symptoms, such as sensorineural hearing loss (SNHL), tinnitus, vertigo, and its clinical significance [1, 3].

To date, many radiological classification criteria based on magnetic resonance imaging (MRI) have been proposed to examine the vascular compression on vestibulocochlear nerve in cerebellopontine angle (CPA) region, such as Chavda [4], Gorrie [5], Kazawa grading systems [6], as well as other classification approaches [7, 8]. Specifically, the Chavda grading system described the depth of extension of the anterior inferior cerebellar artery (AICA) loop into the internal auditory canal (IAC) [4]. Alternatively, Gorrie grading system assessed the extent of contact between the AICA loop and the vestibulocochlear nerve [5]. The recent Kazawa system defined loop formation of AICA or posterior inferior cerebellar artery (PICA) branch and its extension in IAC [6]. Using the above radiological classifications, previous literatures have explored the relationship between neurovascular compression and SNHL, tinnitus or vertigo in unselected patients with non-specific neuro-otologic diagnoses, yielding inconsistent findings [7, 9, 10]. Recently, several studies investigated this relationship in patients with a specific diagnosis. For instance, based on the Chavda and Gorrie grading system, Kim et al*.* found that anatomical variances of the AICA loop position did not affect the incidence of idiopathic sudden SNHL (ISSNHL) or co-morbid symptoms including tinnitus and vertigo [11]. Nevertheless, based on the Kazawa system, Ezerarslan et al*.* showed that some sub-types of ACIA/PICA branching patterns in CPA region are more prevalent in patients with ISSNHL, who are more likely to become refractory to standard therapies than those with other sub-types [12]. This finding implied that the Kazawa grading system might be a promising radiological classification approach to evaluate the association between vascular compression of the vestibulocochlear nerve and audio-vestibular symptoms.

Apart from SNHL, few studies to date have addressed the relationship between vascular compression and vestibular disorders. Based on a grading system derived from Sirikci et al. [13], which primarily assessed the neurovascular contact between AICA and vestibulocochlear nerve, Loader et al*.* found that patients with acute vestibular neuritis with symmetrical caloric reflex had a significantly higher incidence of neurovascular conflict in the CPA region [8]. Recently, in a retrospective study involving unselected patients with various inner ear disorders, based on the vascular loops and vestibulocochlear nerve contact, Beyazal Celikera et al*.* evaluated the coursing pattern of vascular loops in the CPA-IAC region and showed similar MRI findings in patients with and without vertigo [14]. Despite this, other common vestibular disorders, such as Ménière's disease (MD), have not yet been investigated in terms of neurovascular compression.

MD is an idiopathic disorder of the inner ear characterized by repetitive vertiginous episodes, fluctuant SNHL, tinnitus, and aural fullness. The etiology is multifactorial, which may involve excessive endolymph production and decreased endolymph absorption by the endolymphatic sac [15]. Although a previous case report showed the vascular loops in MD patients who were diagnosed clinically [16], neurovascular compression in CPA-IAC is not generally considered as the etiology of MD. To further understand the causal relationship between the vascular compression and audio-vestibular disorders, investigation involving larger sample size is warranted. This retrospective study analyzed Kawaza grading system in patients with unilateral MD with the aim of identifying the radiological features of vascular loop signs in these patients.

## Materials and methods

### Subjects

This retrospective chart review was conducted in Union Hospital affiliated to Tongji Medical College, Huazhong University of Science and Technology, Wuhan, China.

One hundred and thirty-seven patients with unilateral definite MD were enrolled between September 2012 and December 2019. For all patients, a thorough history inquiry, otoscopy, neurotological evaluations (audiometry, impedance, videonystagmography, caloric test, etc.) and imaging examination were conducted for differential diagnosis. The diagnosis of MD was established following the diagnostic guidelines of MD outlined by the American Academy of Otolaryngology-Head and Neck Surgery (AAO-HNS) in 1995 [17]. Sixty-nine subjects without audio-vestibular symptoms were enrolled as the control group.

The exclusion criteria were: (1) middle or inner ear anomaly; (2) middle or inner ear infections (otitis media, mastoiditis, labyrinthitis etc.); (3) retro-cochlear lesions (vestibular schwannoma, internal acoustic canal stenosis etc.); (4) having received previous ear surgery or intratympanic injections; (5) head trauma; (6) bilateral MD; (7) systemic diseases; (8) disorders of central nervous system (vestibular migraine, multiple sclerosis, cerebellar infarction, etc.).

This study was conducted according to the tenets of the Declaration of Helsinki. Informed consent was obtained from each patient and control subject. The project was approved by the ethical committee of Tongji Medical College of Huazhong University of Science and Technology.

### Methods

#### Audio-vestibular evaluations

For all MD patients included, pure tone audiogram was performed during the interictal period. Furthermore, some patients received additional audio-vestibular evaluations, including the electrocochleogram (EcochG) and caloric test. Within 48 h before testing, all subjects were instructed to refrain from alcohol, caffeine or medications that would affect the results of vestibular tests, for instance, sedatives or anti-depressants.

The clinical stage of MD was determined, based on hearing threshold average of the affected side, according to the AAO-HNS guidelines (1995) [17]. EcochG and caloric test were performed as described by previous literature [18]. For EcochG, summating potential (SP) and action potential (AP) were recorded, and the SP/AP ratio was calculated. The SP/AP ratio ≥ 0.4 was deemed as positive. During caloric test, the maximum slow phase velocity (SPV_max_) of caloric nystagmus was measured following each air irrigation, and the canal paresis (CP) was calculated following the Jongkees’ formula. The interaural asymmetry of the caloric nystagmus ≥ 25% was considered abnormal. According to the published criteria [19], if the summated SPV_max_ of the induced nystagmus was < 20°/s after 4 air irrigations, the caloric response is believed to indicate bilateral vestibular hypofunction. In this case, ice water irrigation (4℃, 1.0 ml) would be used to confirm the caloric unresponsiveness.

#### Radiological evaluations

All MRI examination were conducted using the Verio or Magnetom Trio 3 T scanners (Siemens, Erlangen, Germany) with a 12-element phased array coil. Among the routine IAC MR imaging sequence, three-dimensional sampling perfection with application optimized contrasts using different flip angle evolutions (3D-SPACE) was used, (1) to examine the anatomical configurations of AICA/PICA, (2) to exclude inner ear malformation, retro-cochlear pathology and lesions in the CPA. The parameters for the 3D-SPACE sequence were: repetition time (TR), 1000 ms; echo time (TE), 135 ms; slice thickness, 0.5 mm; field of view (FOV), 200 × 200 mm; matrix, 384 × 384; average,2; bandwidth, 289 Hz/Px.

All MRI data were transferred to the workstations and imaging analyses were performed on a picture archiving and communication system (PACS). Radiological data were intermixed and reviewed by two senior neuroradiologists (L.P with an experience of over ten years and L.J over five years) who were blinded to the clinical data. In this study, the Kazawa classification systems were adopted: type IA: non-loop AICA/PICA in the CPA cistern; type IB: non-loop AICA/PICA entering the IAC; type IIA: loop type AICA/PICA in the CPA cistern; and type IIB: loop type AICA/PICA entering the IAC [6]. Supplementary Figs. 1–4 demonstrated typical examples of the branching patterns of AICA/PICA evaluated by Kazawa grading systems.

### Statistics analysis

Statistical analyses were performed by using software SPSS (version 26.0.0.2). All continuous variables are presented as means ± standard deviations (SD) or median and interquartile range (IQR 25th–75th percentiles) after verification of normal distribution. Categorical variables are presented as counts and percentages. Data were tested for normal distribution using the Kolmogorov–Smirnov test. The McNemar-Bowker test was used for the categorical variable comparison between the MD-affected and non-affected side. The distribution of AICA/PICA anatomical variations in different symptoms and hearing outcomes were compared using Chi-square test, Fisher's precision probability test, One-Way ANOVA and Kruskal–Wallis *H* test. The interobserver agreement on radiological assessment was estimated using kappa value. The level of agreement was generally recognized as follows: poor, less than 0.20; fair, 0.21–0.40; moderate, 0.41–0.60; substantial, 0.61–0.80; and almost perfect, 0.81–1.0. The criterion for statistical significance was set at *p* < 0.05.

## Results

The present study enrolled 137 patients with a median age of 51 (42, 58) years, of whom 59 (43.1%) were males and 78 (56.9%) were females. Sixty-nine cases were included in the control group, 25 of which were male (36.2%) and 44 female (63.8%), with a median age of 55 (50, 62) years.

The interobserver agreement on radiological assessment was almost perfect (kappa value = 0.861). Therefore, the results evaluated by one neuroradiologist were used randomly for further analyses.

According to Kazawa grading system, among the MD-affected ears, 6 cases (4.4%) were classified as type IA, 27 cases (19.7%) type IB, 60 cases (43.8%) type IIA, and 44 cases (32.1%) type IIB. Meanwhile, among the MD non-affected ears, 8 cases (5.8%) were categorized as Kazawa type IA, 28 cases (20.4%) type IB, 71 cases (51.8%) type IIA, and 30 cases (21.9%) type IIB. There was no significant interaural difference in the distribution of Kazawa types for patients with unilateral MD (${x}^{2}$ = 4.737, *p* = 0.578). In the control group (69 subjects and 138 ears), 8 ears (5.8%) met the criteria of Kazawa type IA, 29 ears (21%) type IB, 69 ears (50%) type IIA, and 32 ears (23.2%) type IIB. The difference in radiological grading was not significant when comparing the MD-affected ears and the ears of control subjects (${x}^{2}$ = 2.876, *p* = 0.411).

Based on the level of hearing loss, 13 (9.5%) MD patients met the criteria of stage I, 29 (21.2%) patients stage II, 73 (53.3%) patients stage III, and 22 (16.1%) patients stage IV. There was no significant correlation between Ménière’s stage and Kazawa staging of the affected side in unilateral MD patients (*H* = 2.679, *p* = 0.444).

The PTA of the MD patients with Kazawa type IA in the affected ears was 42.5 ± 15.6 dB, those with type IB 49.6 ± 17.5 dB, those with type IIA 53.3 ± 20.2 dB, and those with type IIB 50.1 ± 19.4 dB. No significant correlation was found between the PTA and Kazawa staging of the MD-affected ears (*F* = 0.750, *p* = 0.524).

A total of 83 MD patients underwent the EcochG. Negative result was yielded in 25 MD-affected ears, which comprised 2 ears with type IA, 4 ears with type IB, 12 ears with type IIA and 7 ears with type IIB. Fifty-eight MD-affected ears showed positive result, and ears with type IA, IB, IIA and IIB were 3, 13, 27 and 15, respectively. Kazawa staging in CPA-IAC region did not affect EcochG findings in the MD-affected ears (${x}^{2}$ = 0.827, *p* = 0.867). Furthermore, the SP/AP ratio of the MD-affected ears was 0.63 ± 0.34 in Kazawa type IA, 0.81 (0.49, 1) in IB, 0.74 (0.31, 1) in IIA, and 0.77 (0.31, 1) in IIB. No significant correlation was identified between the SP/AP ratio and Kazawa staging of the affected ear in patients with unilateral MD (*H* = 0.874, *p* = 0.832).

A total of 121 MD patients underwent caloric test. Caloric response was normal in 57 cases, which comprised 2 cases with type IA, 13 cases with type IB, 29 cases with type IIA and 13 cases with type IIB. Abnormal caloric result was identified in 64 cases, which comprised 3 cases with type IA, 11 cases with type IB, 25 cases with type IIA and 25 cases with type IIB. There was no significant correlation between Kazawa staging in CPA-IAC region and caloric findings in the affected ears of patients with unilateral MD (${x}^{2}$ = 4.116, *p* = 0.248). Moreover, the CP value of the MD patients with Kazawa type IA in the affected ears was 41.4% ± 38.3%, those with type IB 22.5% (7%, 51.5%), those with type IIA 23% (7.5%, 41%), and those with type IIB 36.4% ± 24.3%. There was no significant correlation between the CP value and Kazawa staging of the affected ear in patients with unilateral MD (*H* = 2.984, *p* = 0.394).

## Discussion

The present study showed no statistical difference in the distribution of the Kazawa types in the MD-affected ear, MD non-affected ears and control ears. And the Kazawa type IIA was the most prevalent type in all three subgroups, constituting 43.8%, 51.8% and 50% in the MD-affected, MD non-affected and control ears, respectively. Using three-dimensional fast imaging employing steady-state acquisition (3D-FIESTA) sequence in patients with ISSNHL, Ezerarslan et al*.* found that the most common type was Kazawa type IIB (35.3%) in the study group and type IIA (40.8%) in the control subjects [12]. In this study, 3D-SPACE sequences were adopted, and our results were slightly different from those of the above study. Our findings suggested that the Kazawa system-based MRI measurement cannot discriminate the affected ears of unilateral MD patients from the healthy ears, indicating that the presence of vascular loops in the CPA-IAC could be considered as natural anatomical variation. Currently, vestibular disorders are primarily diagnosed based on clinical manifestations, and neurotological examinations could provide useful information to complement this clinical diagnosis. The presence of vascular loop in the CPA-IAC region is not included in any diagnostic criteria for vestibular disorders except for vestibular paroxysmia (VP) [20], in which the neurovascular compression has been considered as the underlying mechanism. In fact, due to the lack of objective diagnostic test, vascular compression is a diagnosis of exclusion and patients with this syndrome are often misdiagnosed [3, 5]. The causal relationship between audio-vestibular symptom and the presence of vascular loops in CPA-IAC region is still controversial. Most of the studies addressing this issue recruited patients with unspecific audio-vestibular symptoms. Only a few studies enrolled patients with a definite diagnosis, such as ISSNHL, vestibular neuritis, or VP [8, 11, 21]. Two pathophysiological mechanisms have currently been hypothesized to explain vascular-cranial nerve compression syndrome: focal demyelination and vascular insufficiency [9, 10]. Although the vascular compression syndromes have been considered as a major cause of hemifacial spasm and trigeminal neuralgia, its role in audio-vestibular symptoms remains debatable.

In this study, the presence of vascular loops in CPA-IAC region did not associate with the Ménière’s stage or the results of EcochG in MD-affected ears. The clinical MD stage is determined by hearing level and reflects the severity of cochlear damage in the affected ear. SP/AP ratio is considered as a functional indicator of cochlear endolymphatic hydrops (ELH), which are the pathological hallmark of MD. Therefore, our results suggest that vascular loops in CPA-IAC region are irrelevant to the severity of cochlear impairment in MD patients. It is universally agreed that ELH, the histopathological hallmark of MD, is caused by disturbed homeostasis of the inner ear [15]. Cerebrovascular circulation irregularities are assumed to be involved in the development of ELH and MD. Histologically, the number of vessels in the stria vascularis in ears with MD was found to be smaller than those in normal controls in all cochlear turns [22]. And occlusion of the vein of the vestibular aqueduct was shown in patients with severe MD [23]. Clinical observations also support vascular insufficiency theory, such as peak age of onset, concomitant diseases and the effectiveness of betahistine in relieving symptoms [24]. Chronic venous insufficiency could also contribute to ELH in patients with MD [25]. Several recent studies have suggested a potential association between the chronic cerebrospinal venous insufficiency (CCSVI) and MD [25–27], despite some controversies. In addition, venous stasis may be considered as a further pathophysiological mechanism underlying MD [26]. Our study mainly focused on the arterial system and found no correlation between AICA/PICA loops in the CPA-IAC region and the clinical features of MD, indicating further investigations might be warranted to explore the clinical relevance of cerebrospinal venous insufficiency rather than arterial system in the pathogenesis of MD [26].

The current study also demonstrated that the presence of vascular loops in CPA-IAC region did not associate with the results of caloric test in patients with unilateral MD. To date, few studies have focused on this association in patients with vertigo or dizziness. Applebaum and Valvassori described normal caloric response in 50% of a small cohort of SNHL patients with vascular loop, which raised doubts about the causality between audio-vestibular symptoms and vascular loops [28]. Recently, the relationship between the caloric reflex and vascular loops has been studied in acute vestibular syndrome. A significantly higher number of neuro-vascular conflicts was observed in patients presenting with symptoms of acute vestibular neuritis with normal caloric response, compared to those with abnormal caloric results and controls, suggesting a possible connection between vascular loops and acute dysfunction in vestibular system [8]. As for episodic vestibular syndrome, it has been recognized that the pathogenesis of VP involves chronic contact between the nerve and blood vessel [21]. Using constructive interference in steady state (CISS) MRI, Hüfner et al*.* found most VP patients had at least one site of neurovascular compression, and no strong systematic agreement between the side of neurovascular compression and the vestibular deficit, which were measured by caloric test and hyperventilation induced nystagmus [21]. Caloric test is a traditional vestibular test, mainly evaluating the vestibular ocular reflex (VOR) function of horizontal semicircular canal using non-physiological stimulus within the frequency range of 0.002–0.004 Hz. And the CP value may be related to the severity of ELH and the degree of vestibular hair cell impairment in patients with MD [19]. This study, for the first time, showed no associations between caloric response and vascular loops in CPA-IAC area in patients with MD, a prototype of episodic vestibular syndrome. Therefore, our findings suggested that vascular loops in CPA-IAC region did not affect low-frequency angular VOR function in patients with unilateral MD. Whether vascular loops may affect the functional status of otolith-collic/ocular reflex (measured by vestibular myogenic vestibular potentials) or the high-frequency angular VOR (measured by video head impulse test) in other vestibular disorders requires further studies.

The application of MRI has provided novel insight for the pathogenesis of MD. High-resolution MRI with gadolinium as the contrast agent allows direct visualization of ELH in the inner ear, including the vestibular and cochlear compartment, which can facilitate diagnosis of MD [29, 30]. Moreover, non-contrast MRI is also used in MD, mainly for indirect evaluation of the size of the endolymphatic sac or endolymphatic drainage system [31], evaluation of bidirectional vestibulocochlear nerves diameters and cross-sectional areas [32], and others. To the best of our knowledge, this study is the first imaging study assessing neurovascular compression in the CPA-IAC region in a large cohort of MD patients. Our results showed that imaging findings based on the Kazawa classification system did not correlate with the lateralization, Ménière’s stage, EcochG, and caloric test findings in unilateral MD, which is in line with the results of a systematic review. Papadopoulou et al. included 15 studies for systematic review and found that, the radiological results did not consistently correlate with audio-vestibular symptoms in about 70% of the included patients, suggesting that vascular loops may be an anatomical variant in a significant majority of cases [3]. Of note, the etiologies underlying these audio-vestibular symptoms were undefined in most cases. This also calls for the development of new radiological grading systems to better understand the role of neurovascular compression in patients with audio-vestibular symptoms, although a wide variety of classification systems are already available.

This retrospective study was subject to certain limitations. Firstly, our routine diagnostic workup for patients with unilateral MD does not include gadolinium enhanced MRI of the inner ear. Secondly, this study mainly addressed the correlation between the AICA configuration in IAC-CPA region and the clinical features and audio-vestibular results of unilateral MD. It is reported that the depth of extension in IAC or the extent of neurovascular contact of AICA loops may also affect the audio-vestibular symptoms [5, 7]. Future studies involving other MRI-based grading systems, such as Chavda and Gorrie criteria, are warranted.

## Conclusions

In patients with unilateral MD, the radiological presence of vascular loops in CPA-IAC region did not correlate with the laterality, clinical stage, the results of EcochG and caloric test, suggesting that this presence may be a natural anatomical variation for patients with unilateral MD.

## Supplementary Information

Below is the link to the electronic supplementary material.

Supplementary Fig. 1 3D-SPACE MRI images of a 53-year-old female with left-side unilateral MD. (a-h) axial, high-resolution, T2-weighted MRI scan showing Kazawa classification type IA in which non-loop AICA/PICA (arrow) in the CPA cistern

Supplementary Fig. 2 3D-SPACE MRI images of a 56-year-old female with left-side unilateral MD. (a-f) axial, high-resolution, T2-weighted MRI scan showing Kazawa classification type IB in which non-loop AICA/PICA (arrow) extending into the IAC

Supplementary Fig. 3 3D-SPACE MRI images of a 49-year-old female with left-side unilateral MD. (a-p) axial, high-resolution, T2-weighted MRI scan showing Kazawa classification type IIA in which loop type AICA/PICA (arrow) in the CPA cistern

Supplementary Fig. 4 3D-SPACE MRI images of a 45-year-old female with left-side unilateral MD. (a-j) axial, high-resolution, T2-weighted MRI scan showing Kazawa classification type IIB in which loop type AICA/PICA (arrow) extending into the IAC

## Data Availability

The original contributions presented in the study are included in the article and supplementary material, further inquiries can be directed to the corresponding authors.
