# Supplementary figures and images for: Radiological presence of vascular loops in the cerebellopontine angle region in patients with unilateral Ménière’s disease
Source: Eur Arch Otorhinolaryngol. 2023 Feb 3;280(7):3195–201. doi: 10.1007/s00405-023-07838-9 (PMC10219856; doi:10.1007/s00405-023-07838-9)

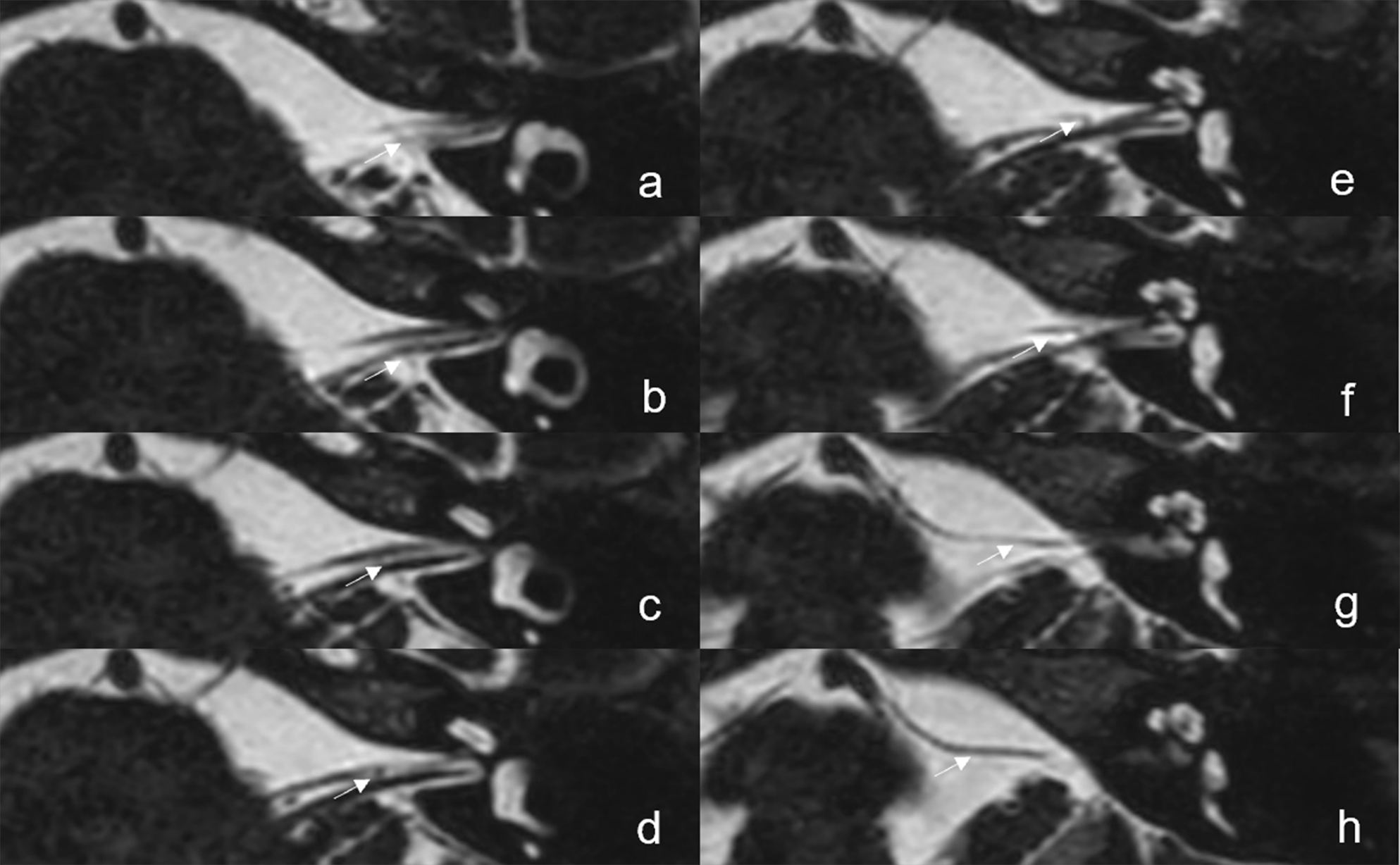

Supplement: Supplementary file 1 — Supplementary Fig. 1 3D-SPACE MRI images of a 53-year-old female with left-side unilateral MD. (a-h) axial, high-resolution, T2-weighted MRI scan showing Kazawa classification type IA in which non-loop AICA/PICA (arrow) in the CPA cistern [file 405_2023_7838_MOESM1_ESM.tif]

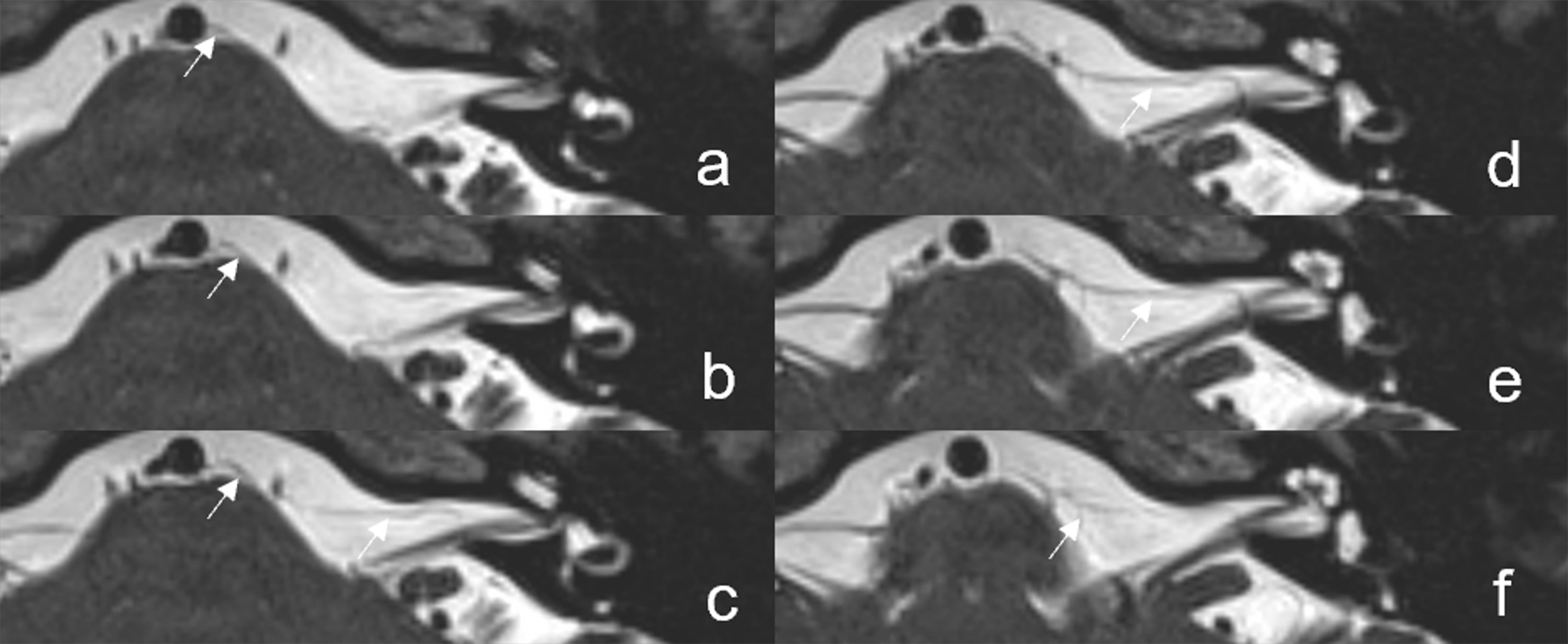

Supplement: Supplementary file 2 — Supplementary Fig. 2 3D-SPACE MRI images of a 56-year-old female with left-side unilateral MD. (a-f) axial, high-resolution, T2-weighted MRI scan showing Kazawa classification type IB in which non-loop AICA/PICA (arrow) extending into the IAC [file 405_2023_7838_MOESM2_ESM.tif]

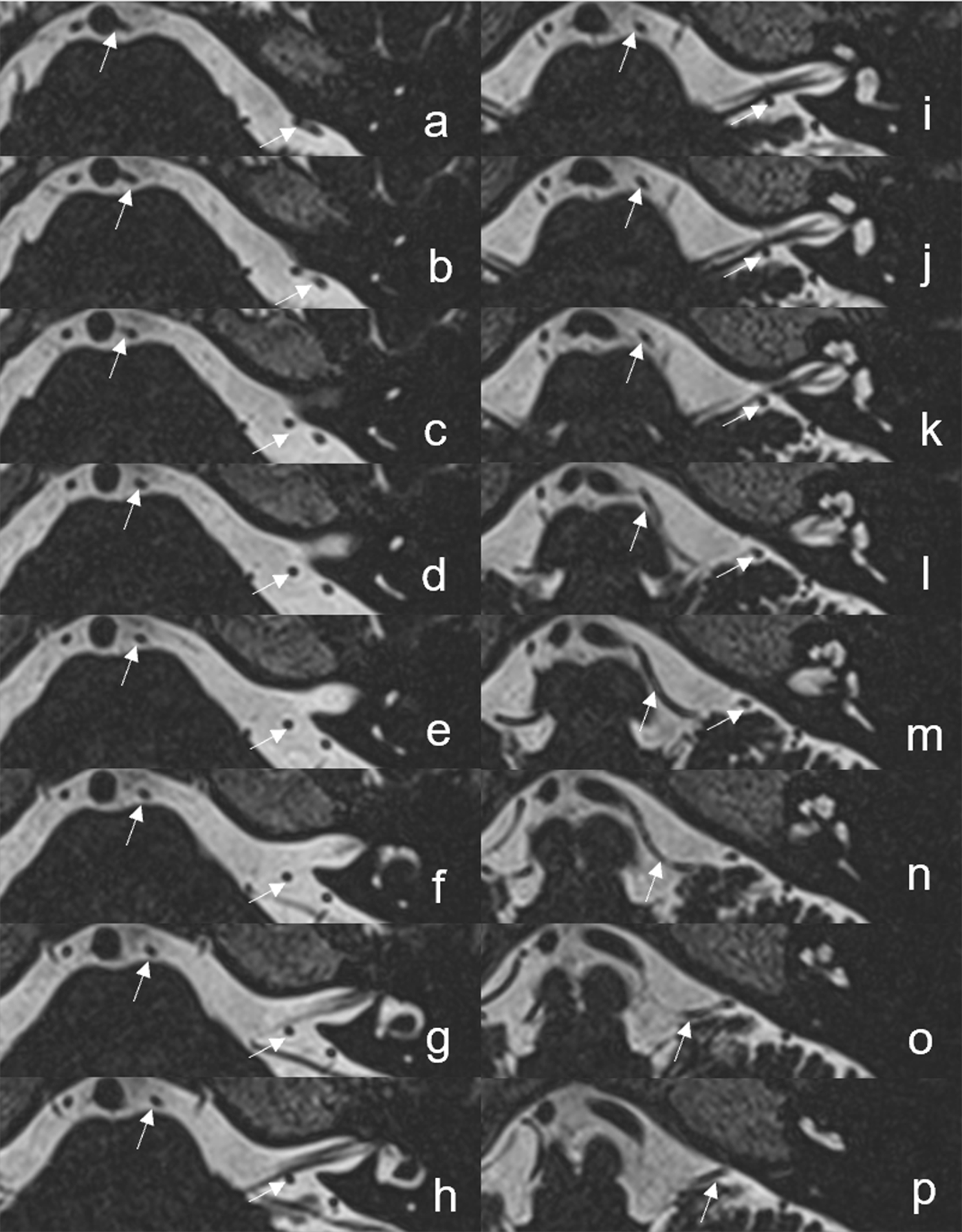

Supplement: Supplementary file 3 — Supplementary Fig. 3 3D-SPACE MRI images of a 49-year-old female with left-side unilateral MD. (a-p) axial, high-resolution, T2-weighted MRI scan showing Kazawa classification type IIA in which loop type AICA/PICA (arrow) in the CPA cistern [file 405_2023_7838_MOESM3_ESM.tif]

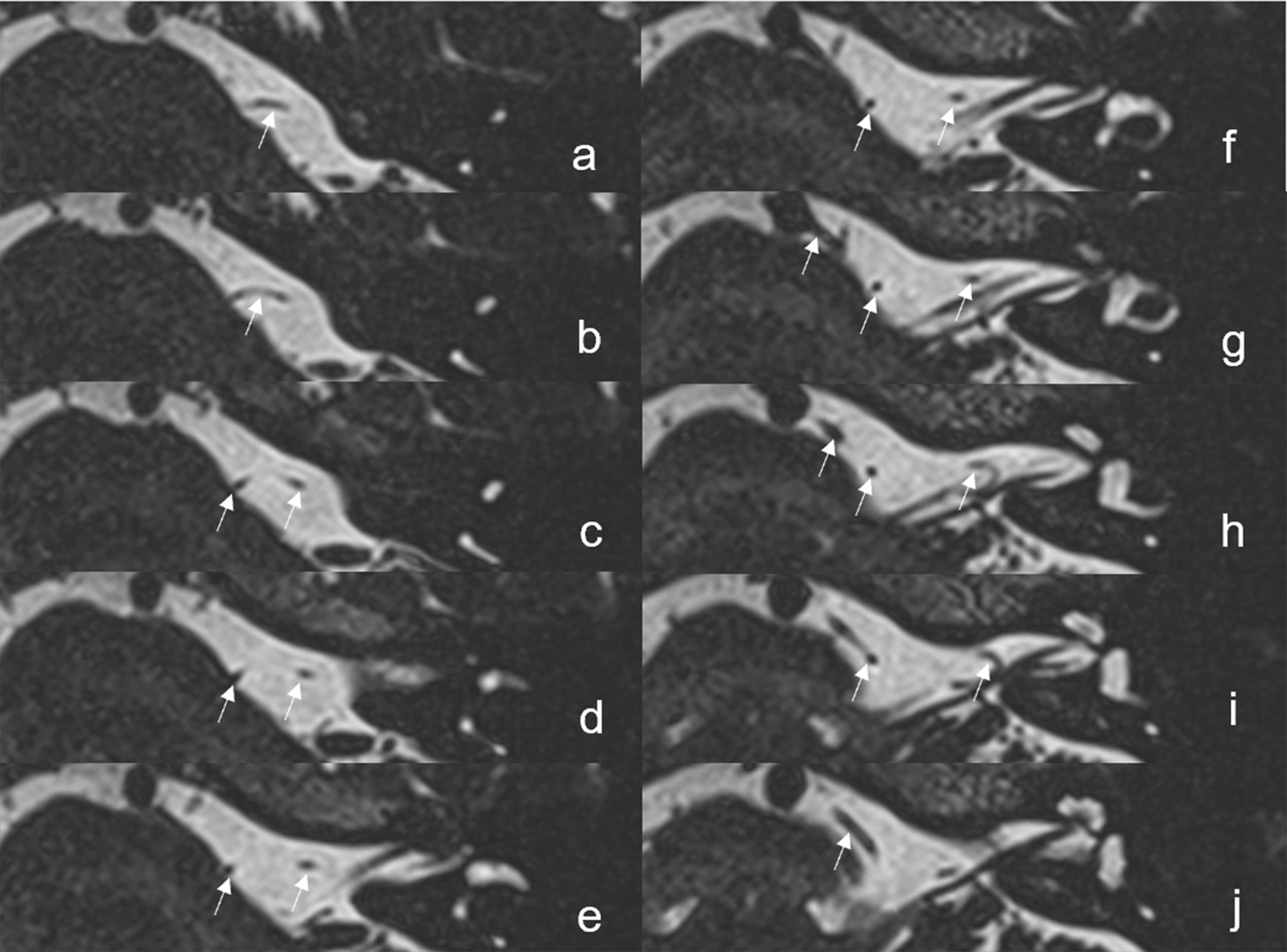

Supplement: Supplementary file 4 — Supplementary Fig. 4 3D-SPACE MRI images of a 45-year-old female with left-side unilateral MD. (a-j) axial, high-resolution, T2-weighted MRI scan showing Kazawa classification type IIB in which loop type AICA/PICA (arrow) extending into the IAC [file 405_2023_7838_MOESM4_ESM.tif]
